# A longitudinal analysis of the relationship between serum uric acid and residual renal function loss in peritoneal dialysis patients

**DOI:** 10.1080/0886022X.2020.1761387

**Published:** 2020-05-13

**Authors:** Chiehlun Yang, Xinxin Ma, Wenbo Zhao, Yanru Chen, Hongchun Lin, Dan Luo, Jun Zhang, Tanqi Lou, Yu Peng, Hui Peng

**Affiliations:** a Nephrology Division, Department of Medicine, The Third Affiliated Hospital of Sun Yat-Sen University, Guangzhou, Guangdong, China; b Nephrology Division, Department of Medicine, Guangdong Province Traditional Chinese Medical Hospital, Guangzhou, Guangdong, China

**Keywords:** Peritoneal dialysis, uric acid, residual renal function, end-stage renal disease

## Abstract

**Background:**

Hyperuricemia occurs frequently in patients with continuous ambulatory peritoneal dialysis (CAPD). This study aimed to evaluate the impact of serum uric acid (UA) over time on residual renal function (RRF) loss in a cohort of patients with CAPD.

**Methods:**

A total of 201 patients who started CAPD therapy between January 1, 2008 and April 30, 2016 were included in this single-center, retrospective cohort study. All patients were followed up until December 31, 2016. The median follow-up time was 23.43 ± 16.60 months. RRF loss was represented as the time to anuria.

**Results:**

Eighty-six patients developed anuria within 5 years. Multivariate Cox regression analysis showed that time-averaged serum UA and peritonitis were independent risk factors for RRF loss, while weekly Kt/V urea was a protective factor. Cox proportional hazard regression models showed that both patients with time-averaged uric acid (TA-UA) < 6.77 mg/dL [hazard ratio (HR) = 1.165, 95% confidence interval (CI) 1.054–1.387; *p* < 0.05] and those with TA-UA≥ 7.64 mg/dL (HR = 1.184, 95% CI 1.045–2.114; *p* < 0.05) had a higher risk of RRF than those with TA-UA in the range of 6.77–7.64 mg/dL. Penalized spline smoothing also showed a U-shaped relationship between continuous UA and RRF loss.

**Conclusion:**

The present study demonstrated that both high and low serum UA over time were associated with RRF loss in patients with CAPD.

## Introduction

Uric acid (UA) is the final product of purine metabolism caused by xanthine oxidase or xanthine dehydrogenase in humans [1]. The kidneys play an important role in the excretion of UA [2]. In the general population, hyperuricemia increases the risks of chronic kidney disease (CKD), end-stage renal disease (ESRD), and cardiovascular mortality [3–7]. A recent study has reported that even in healthy individuals, high UA level is associated with nephron loss together with age and female gender, leading to decline in total glomerular filtration rate (GFR) [8]. However, in CKD patients, whose UA level increases with deterioration of kidney function and decline in renal clearance of UA, the effect of UA on kidney disease progression remains controversial. A study of patients of CKD stage 3–4 found that hyperuricemia may be an independent risk factor for all-cause and CVD mortality, but not CKD progression [9]. Some other studies suggested that UA is associated with progression of kidney disease in CKD patients [10–13]. This inconsistency can be due to differences in CKD severity, underlying causes of CKD such as diabetes and the presence of other confounding factors.

The clinical impact of UA on residual renal function (RRF) in patients treated with peritoneal dialysis (PD) is more complicated. Only a handful of studies have investigated the association between UA and RRF in patients with ESRD who were treated with PD [14,15]. Previous studies measured baseline UA, but scant attention has been directed toward UA during follow-up. Compared with baseline UA, time-dependent UA is a more comprehensive reflection of the changes of serum UA levels over time in the clinical course of kidney disease progression. Thus, we attempted to evaluate the effect of longitudinal serum UA on RRF loss in patients undergoing continuous ambulatory peritoneal dialysis (CAPD), which may provide insights into the preservation of RRF.

## Materials and methods

### Patients recruitment and study design

This retrospective observational cohort study was conducted at a single medical center in China. The study population included patients who began CAPD therapy between January 1, 2008 and April 30, 2016 in the Third Affiliated Hospital of Sun Yat-sen University (Guangzhou). All patients over 18 years of age were recruited with no limitation of gender. Exclusion criteria for the study were as follows: (1) presence of anuria (24-h urine volume <100 mL) before starting PD (*n* = 17), (2) duration of maintenance PD less than 3 months (*n* = 17), (3) prior kidney transplantation (*n* = 2), and (4) inadequate data (*n* = 28). The study was reviewed and approved by the Institutional Review Board of the Third Affiliated Hospital of Sun Yat-sen University (No. 02-226-01). Informed consent forms were signed by the subjects, confirming their decision of being informed and voluntary participation in the study. Informed consents by all participants were obtained before enrollment.

### Data collection

Baseline data were collected within 1 month of the initiation of PD therapy and all patients were followed up every 3 months thereafter. Demographic and clinical information, including gender, age, body mass index, causes of CKD, history of diabetes, and history of cardiovascular disease (CVD), was collected at the beginning of PD. Laboratory parameters were collected during the observational period, including hemoglobin, albumin, blood urea nitrogen, creatinine, calcium, phosphorus, cholesterol, triglyceride, bicarbonate, UA, intact parathyroid hormone (iPTH), ferritin, and C-reactive protein (CRP). Serum UA measured at every visit during the follow-up was calculated as time-averaged UA (Time-averaged value = (N1 + N2 + N3 + …. + Nn)/*n*). PD-related parameters were also collected, including RRF, total Kt/V urea, Kt/V urea for PD, Kt/V urea for RRF, dialysate to plasma ratio (D/P) at 4 h, and peritonitis rate. CVD was defined as having one or more of the following history: stable angina, unstable angina, myocardial infarction, percutaneous coronary intervention, coronary artery bypass grafting, heart failure, or stroke. RRF was calculated as the mean of the urea and creatinine clearance based on a 24-h urine collection, adjusted for body surface area. Body surface area was calculated using the Gehan and George equation. And RRF was calculated by the following formula:

RRF=12[UrineCr(μmol/L)SerumCr(μmol/L)+UrineUrea(mmol/L)SerumUrea(mmol/L)]×UrineVolume(mL)1440

Medication records during the follow-up period were also collected according to electronic prescriptions information and patients’ medical records in visits. For each medical assessment, either face-to-face interviews or nurse-led telephone follow-up were conducted by certified PD nurses, to evaluate the PD patients’ general conditions and concomitant medications.

The primary outcome in the study was RRF loss, which was represented by the time to first occurrence of anuria. Anuria was defined as a 24-h urine volume less than 100 mL on two consecutive occasions. Study outcomes were documented until December 31, 2016. If clinical events like switching to hemodialysis, kidney transplantation, loss to follow-up, or death occurred in CAPD patients during follow-up period, their data were censored at the time of switching to other modalities or events.

### Statistical analysis

Summary statistics are presented as mean ± standard deviation for continuous variables with a normal distribution, or as number (*n*) and percentage (%) for categorical variables, unless otherwise indicated. The Kolmogorov–Smirnov normality test was used to determine whether continuous variables were normally distributed.

To identify independent risk factors of RRF loss during the follow-up period, all significant (*p* ≤ 0.05) variables in univariate analyses were further included in the multivariable Cox regression analysis. Subsequently, patients were classified according to time-averaged uric acid (TA-UA; mg/dL) tertiles: <6.77, lower tertile; 6.77–7.64, middle tertile; and ≥7.64, upper tertile. Clinical parameters of the three groups were compared to find out whether there is a difference. Continuous data with a normal distribution were compared by analysis of variance; skewed continuous data were compared by the Kruskal–Wallis test. Competing risk mixture models were further performed to analyze the impact of TA-UA on the appearance of RRF loss. The results are shown as hazard ratios (HRs) and 95% confidence intervals (CIs).

To strengthen our analysis, Cox regression and penalized spline smoothing were used to assess the association between risk of RRF loss and time-varying UA levels. The time-varying variable was calculated by the following formula: time-varying value = (mean serum value – baseline value)/baseline value × 100%. Thus, negative and positive values indicated decreases and increases from baseline values, respectively. *p-*values less than 0.05 were considered statistically significant. Statistical analysis was performed using SPSS for Windows software (version 22.0; SPSS Inc., Chicago, IL) and R software (version 3.3.2; R Foundation for Statistical Computing, Vienna, Austria).

## Results

### Demographic and clinical characteristics of the study population

**Figure 1.**
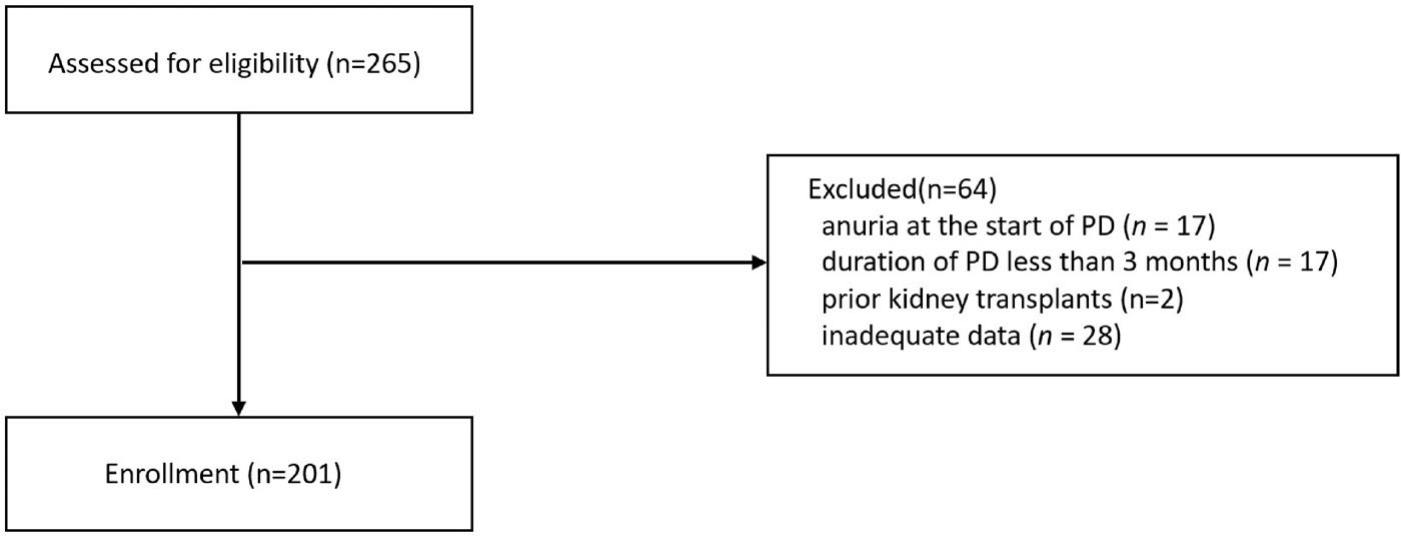
Flow chart of patient selection.

We identified 265 CAPD patients who fulfilled the inclusion criteria. Sixty-four PD patients were excluded from the analysis in accordance with the exclusion criteria, and consequently a total of 201 patients were included (Figure 1). Among patients enrolled, 21 (10.45%) patients transferred to hemodialysis or other dialysis centers, 21 (10.4%) underwent renal transplantation, 29 (14.4%) died, and 130 (64.7%) remained on CAPD (Figure 1). The baseline demographic and clinical statistics of the cohort are presented in Table 1, together with time-averaged values and medications used during follow-up period. The mean age of the patients was 48.32 ± 14.16 years and 60.7% of them were male (*n* = 122). Chronic glomerulonephritis (58.21%) was the most common cause of ESRD in these patients. Meanwhile, a higher percentage of CVD was observed in male patients. The mean UA level was 7.13 ± 3.26 mg/dL at baseline, with no gender difference. Only 10.5% of the study population received UA-lowering therapy at baseline. During the follow-up period of 23.43 ± 16.60 months, Eighty-six patients (42.8%) out of the 201 patients progressed to anuria. The blood pressure level, serum creatinine, and BUN level in all patients was significantly decreased during follow-up compared with baseline. However, TA-UA in both male and female patients was higher than baseline UA. Also, there was a significant increase in usage of urate-lowering drug, diuretics and RAS inhibitors during follow-up (Table 1).

**Table 1.** Demographic and clinical characteristics.

Characteristic	Total (baseline)	Male	Female	Total (time average)	Male	Female
Number of patients	201	122	79	—	—	—
Age (year)	48.32 ± 14.16	47.84 ± 14.27	49.99 ± 13.84	—	—	—
Dialysis duration (month)	—	—	—	23.43 ± 16.60	21.46 ± 18.01	24.91 ± 15.54
BMI (kg/m^2^)	22.21 ± 4.27	22.26 ± 3.88	22.19 ± 4.47	22.39 ± 4.12	22.36 ± 3.82	22.42 ± 4.45
Etiology of CKD (%)						
Chronic glomerulonephritis	117 (58.21)	70 (57.38)	47 (59.49)	—	—	—
Diabetic kidney disease (%)	42 (20.9)	26 (21.31)	16 (20.25)	—	—	—
Hypertension	9 (4.48)	6 (4.91)	3 (3.80)	—	—	—
Others	33 (16.42)	20 (16.39)	13 (16.46)	—	—	—
Comorbidity						
Diabetes (%)	55 (27.36)	32 (26.23)	23 (29.11)	—	—	—
Cardiovascular/cerebrovascular disease (%)	70 (34.83)	48 (39.34)	22 (27.85)	—	—	—
Systolic blood pressure (mmHg)	161.20 ± 24.95	164.34 ± 25.67	156.35 ± 23.12	141.62 ± 26.21^&^	143.29 ± 28.62*	139.66 ± 27.54^#^
Diastolic blood pressure (mm Hg)	92.72 ± 16.77	93.98 ± 16.86	90.77 ± 16.55	90.63 ± 15.37	91.01 ± 16.26	89.5 5 ± 13.72
PD-related parameters						
Total Weekly Kt/V urea	2.21 ± 0.08	2.22 ± 0.09	2.19 ± 0.06	2.13 ± 0.09^&^	2.10 ± 0.07*	2.15 ± 0.11^#^
KT/V urea for PD	1.55 ± 0.04	1.53 ± 0.03	1.56 ± 0.03	—	—	—
KT/V urea for RRF	0.66 ± 0.04	0.69 ± 0.06	0.63 ± 0.03	—	—	—
D/P at 4 hours	0.66 ± 0.16	0.68 ± 0.14	0.65 ± 0.18	0.60 ± 0.24	0.56 ± 0.19	0.62 ± 0.28
Urine volume(mL/d)	1287.69 ± 624.40	1346.70 ± 681.54	1196.57 ± 515.03	—	—	—
RRF (mL/min per 1.73m^2^)	5.36 ± 3.05	5.88 ± 3.26	4.68 ± 2.57	—	—	—
Peritonitis rate (episodes per patient year)	—	—	—	0.19	0.20	0.17
Laboratory values						
Uric acid (mg/dL)	7.13 ± 3.46	7.12 ± 3.53	7.14 ± 3.32	7.24 ± 4.52^&^	7.22 ± 4.23*	7.26 ± 4.92^#^
BUN (mmol/L)	30.90 ± 14.68	30.35 ± 12.69	31.78 ± 15.41	26.88 ± 17.41^&^	28.25 ± 18.53*	23.74 ± 14.21^#^
Creatinine (mg/dL)	11.04 ± 3.80	10.71 ± 3.53	11.56 ± 4.11	9.47 ± 2.84^&^	9.23 ± 2.52*	10.16 ± 3.11
Calcium (mg/dL)	8.20 ± 1.18	8.18 ± 1.15	8.25 ± 1.20	10.19 ± 1.40	10.15 ± 1.35	10.23 ± 1.66
Phosphorus (mg/dL)	6.39 ± 1.55	6.34 ± 1.76	6.46 ± 1.46	6.32 ± 1.38	6.29 ± 1.28	6.33 ± 1.46
Bicarbonate (mEq/L)	19.08 ± 5.82	18.97 ± 4.04	19.26 ± 6.86	20.18 ± 4.86	19.97 ± 3.86	20.35 ± 5.26
Total cholesterol (mmol/L)	4.98 ± 0.37	4.98 ± 0.41	4.99 ± 0.35	4.87 ± 0.43	4.93 ± 0.49	4.84 ± 0.38
LDL-C (mg/dL)	107.88 ± 34.38	108.66 ± 31.86	107.12 ± 36.35	107.89 ± 32.33	108.23 ± 33.23	107.73 ± 31.92
Hemoglobin(g/dL)	8.20 ± 2.70	8.24 ± 2.51	8.15 ± 2.82	10.19 ± 1.40	11.24 ± 1.51	9.15 ± 1.32
CRP (mg/L)	3.61 ± 1.51	3.78 ± 1.72	3.55 ± 1.35	4.40 ± 1.30	4.78 ± 1.52	4.35 ± 1.15
Medications						
Urate-lowering drugs (%)	21 (10.53)	15 (12.30)	6 (0.08)	52 (25.87)^&^	36 (29.51)*	16 (20.25)^#^
Diuretics (%)	86 (42.79)	49 (40.16)	37 (46.84)	111 (55.22)^&^	65 (53.28)*	46 (58.23)^#^
RAS inhibitor (%)	138 (68.66)	83 (68.03)	55 (69.62)	182 (90.55)^&^	113 (92.26)*	69 (87.34)^#^

Data are expressed as mean ± SD or number (percentage). BMI: body mass index; BUN: blood urea nitrogen; CRP, C-reactive protein; Kt/V: urea clearance index; LDL-C: low-density lipoprotein cholesterol; RAS inhibitor: Renin-angiotensin system inhibitor.

^&,*,#^*p* < 0.05; ^&^Comparison of baseline and follow-up values among total patients; *Comparison of baseline and follow-up values among male patients; ^#^comparison of baseline and follow-up values among female patients.

### TA-UA was an independent risk factor for RRF loss during PD therapy

Cox regression analysis was performed using time-averaged variables to identify risk factors associated with anuria (Table 2). Univariate Cox analysis indicated that TA-UA, weekly Kt/V urea, bicarbonate level, phosphorous level and history of peritonitis were associated with risk of RRF loss. Factors identified as significant (*p* < 0.05) in univariate analysis were included in the multivariate Cox regression analyses, which showed that TA-UA was an independent risk factor of developing anuria in CAPD patients. In addition, peritonitis was independent risk factors for RRF loss while weekly Kt/V urea was a protective factor (Table 2).

**Table 2.** Predictive factors associated with anuria in Cox regression analysis.

	Univariate			Multivariate		
	HR	95% CI	*p* Value	HR	95% CI	*p* Value
Age	1.333	(0.714,2.003)	0.671	—	—	—
Gender (female versus male)	1.672	(0.847,2.392)	0.971	—	—	—
BMI (kg/m^2^)	1.005	(0.993,1.017)	0.407	—	—	—
Diabetes(yes versus no)	1.882	(0.914, 2.727)	0.884	—	—	—
Cardiovascular/cerebrovascular disease (yes versus no)	1.562	(0.671, 2.344)	0.781	—	—	—
Systolic blood pressure (mmHg)	1.032	(0.921,1.098)	0.098	—	—	—
Diastolic blood pressure (mmHg)	1.065	(0.871,1.077)	0.123	—	—	—
RAS inhibitor	1.073	(0.422,2.728)	0.883	—	—	—
Diuretics	0.821	(0.513,1.313)	0.410	—	—	—
Urate-lowering drugs use	0.448	(0.163,1.237)	0.448	—	—	—
Weekly Kt/V urea	0.240	(0.127,0.456)	<0.001	0.331	(0.164,0.670)	<0.01
peritonitis (yes versus no)	1.012	(1.001,1.033)	<0.001	1.010	(1.001,1.018)	<0.001
Uric acid (mg/dL)	1.021	(1.010,1.032)	<0.01	1.033	(1.001,1.052)	<0.01
Bicarbonate (mEq/L)	1.006	(1.001,1.113)	<0.05	1.212	(0.914,1.716)	0.923
Phosphorus (mg/dL)	1.052	(1.006,1.100)	<0.05	1.076	(0.877,1.318)	0.267
Calcium (mg/dL)	0.820	(0.615,1.094)	0.178	—	—	—
BUN (mmol/L)	1.001	(0.811,1.003)	0.333	—	—	—
Creatinine (mg/dL)	1.002	(0.657,1.560)	0.658	—	—	—
Total cholesterol (mmol/L)	1.007	(1.000,1.014)	0.066	—	—	—
LDL-C (mg/dL)	1.222	(0.631,1.844)	0.937	—	—	—
Hemoglobin (g/dL)	0.978	(0.998,1.001)	0.595	—	—	—
CRP (mg/dl)	1.453	(0.775,1.138)	0.683	—	—	—

All continuous variables were follow-up values. BMI: body mass index; BUN: blood urea nitrogen; CRP: C-reactive protein; Kt/V: urea clearance index; LDL-C: low-density lipoprotein cholesterol; RAS inhibitor: Renin-angiotensin system inhibitor.

### Higher and lower TA-UA levels were associated with an increased risk of RRF loss during PD therapy

The subjects were divided into three groups according to TA-UA (mg/dL) tertiles: lower tertile, <6.77; middle tertile, 6.77–7.64; and upper tertile, ≥7.64. Significant differences among tertiles were observed in the parameters during follow-up, including total weekly Kt/V urea, BUN, creatinine, phosphorus, bicarbonate, percentages of patients used urate-lowering drugs, and diuretics. (Table 3). However, there was a significant difference in the proportion of PD patients who developed anuria during follow-up period (lower vs. middle vs. upper: 46.15 vs. 30.77 vs. 50.75%, *p* = 0.021), and in time to anuria (lower vs. moderate vs. upper: 16.44 ± 9.02 vs. 24.53 ± 17.09 vs. 15.71 ± 9.12 months, *p* = 0.005), among three groups.

**Table 3.** Baseline characteristics of study subjects stratified by time-averaged serum uric acid levels.

	Uric acid < 6.77 (*N* = 67)	6.77 ≤ Uric acid <7.64 (*N* = 67)	Uric acid ≥ 7.64 (*N* = 67)	*P*
Time to aunria(months)	16.44 ± 9.02	24.53 ± 17.09	15.71 ± 9.12	0.005**
sex (male%)	48.80	48.60	46.20	0.136
age(year)	45.77 ± 15.15	48.62 ± 14.53	53.67 ± 12.77	0.113
BMI(kg/m^2^)	21.88 ± 4.00	21.56 ± 4.86	22.50 ± 4.02	0.111
Diabetes (%)	30.85	26.08	28.36	0.326
Cardiovascular disease (%)	34.31	36.92	37.31	0.487
Etiology (%)				
Glomerulonephritis	63.08	56.92	56.72	0.116
Diabetic nephropathy	21.54	21.54	20.90	0.206
Hypertension	2.3	2.4	2.6	0.341
Other	12.31	12.50	16.42	0.124
Medication				
ACE inhibitors or ARBs (%)	64.62	67.69	75.76	0.597
a-block (%)	53.85	39.06	56.06	0.605
β-block (%)	63.08	62.5	66.67	0.371
Calcium channel blockers (%)	80.00	86.56	85.39	0.056
Diuretics (%)	46.15	51.56	43.33	0.086
VDRAs (%)	43.08	56.25	39.39	0.631
PBAs (%)	49.70	40.63	53.03	0.478
Statin (%)	27.69	34.38	18.18	0.094
Uric acid-lowering drug (%)	10.80	10.90	10.60	0.546
PD-related parameters				
Weekly Kt/V urea	2.45 ± 0.94	2.45 ± 0.48	2.21 ± 0.61	0.066
KT/V urea for PD	1.558 ± 0.43	1.58 ± 0.49	1.59 ± 0.39	0.221
KT/V urea for RRF	0.62 ± 0.44	0.72 ± 0.42	0.65 ± 0.51	0.506
D/P at 4 hrs	0.64 ± 0.15	0.66 ± 0.19	0.70 ± 0.15	0.247
ultrafiltration (L/d)	0.29 ± 0.46	0.20 ± 0.50	0.17 ± 0.37	0.343
Hemoglobin(g/dL)	8.20 ± 2.38	8.40 ± 2.85	8.00 ± 2.80	0.400
Albumin(g/dL)	3.49 ± 0.63	3.57 ± 0.75	3.65 ± 0.61	0.445
BUN(mmol/L)	28.24 ± 15.45	29.85 ± 13.60	27.38 ± 12.22	0.055
Creatinine(mg/dL)	9.99 ± 4.34	10.39 ± 4.17	10.45 ± 3.89	0.066
Calcium(mg/dL)	8.36 ± 1.12	8.08 ± 1.11	8.12 ± 1.16	0.233
Phosphorus(mg/dL)	6.18 ± 1.77	6.35 ± 1.86	6.56 ± 1.91	0.052
Cholesterol(mmol/L)	4.50 ± 1.28	4.89 ± 1.40	5.57 ± 1.04	0.483
Triglyceride(mmol/L)	1.63 ± 1.07	1.78 ± 0.78	1.85 ± 1.08	0.440
Bicarbonate(mEq/L)	19.29 ± 3.83	19.98 ± 4.06	18.03 ± 4.65	0.148
Baseline Uric acid(mg/dL)	8.34 ± 3.04	8.42 ± 3.09	8.57 ± 3.10	0.066
iPTH(pg/mL)	441.76 ± 39.33	451.21 ± 37.66	437.02 ± 34.34	0.358
Ferritin(ng/L)	374.95 ± 46.56	311.54 ± 33.24	400.78 ± 41.40	0.093
Initial RRF (L/min/1.73m2)	5.72 ± 1.07	5.27 ± 1.96	5.52 ± 2.02	0.063
CRP(mg/L)	3.4 ± 5.4	3.3 ± 5.6	3.5 ± 5.7	0.683
RRF loss during 5 years (%)	46.15	30.77	50.75	0.021*

BMI = body mass index, DM = diabetes, CVD = cerebrovascular disease, RRF = residual renal function, PBAs, phosphate binding agents; VDRAs = Vitamin D receptor activators.

The middle tertile, which had fewer RRF loss events and longer duration of residual urine than the other, was used as the reference group for further analyses. After adjusting for peritonitis, total KT/V urea (Table 4 left), multivariate analysis showed that the risk of RRF loss increased by 7.8% and 16.4% in the lower tertile (HR = 1.078, 95% CI 1.011–2.545, *p* = 0.035) and upper tertile (HR = 1.164, 95% CI 1.034–1.782, *p* = 0.039), respectively, compared with the middle tertile. Additionally, adjusting for age, gender, urate-lowering drug use, peritonitis, total KT/V urea (Table 4 right), multivariate analysis showed that the risk of RRF loss increased by 16.5% and 18.4% in the lower tertile (HR = 1.165, 95% CI 1.054–1.387, *p* = 0.037) and upper tertile (HR = 1.165, 95% CI 1.045–2.114, *p* = 0.041), respectively, compared with the middle tertile. The risk of RRF loss is highest in CAPD patients with high TA-UA level, followed by those with low TA-UA level.

**Table 4.** Competing risk mixture model of time-averaged uric acid and risk of RRF loss.

	Model 1			Model 2		
Time-averaged uric acid (mg/dL)	HR	95% CI	*p* Value	HR	95% CI	*p* Value
UA < 6.77	1.078	(1.011,2.545)	0.035	1.165	(1.054,1.387)	0.037
6.77 ≤UA < 7.64	1	(reference)	—	1	(reference)	—
UA ≥ 7.64	1.164	(1.034,1.782)	0.039	1.184	(1.045,2.114)	0.041

Model1 (competing risk mixture model) adjusted by peritonitis, total KT/V urea. Model2 (competing risk mixture model) adjusted by age, gender, urate-lowering drug use, peritonitis, total KT/V urea; the 6.77 ≤ UA < 7.64 as the reference group.

### Higher and lower time-varying UA levels were associated with an increased risk of RRF loss during PD therapy

**Figure 2.**
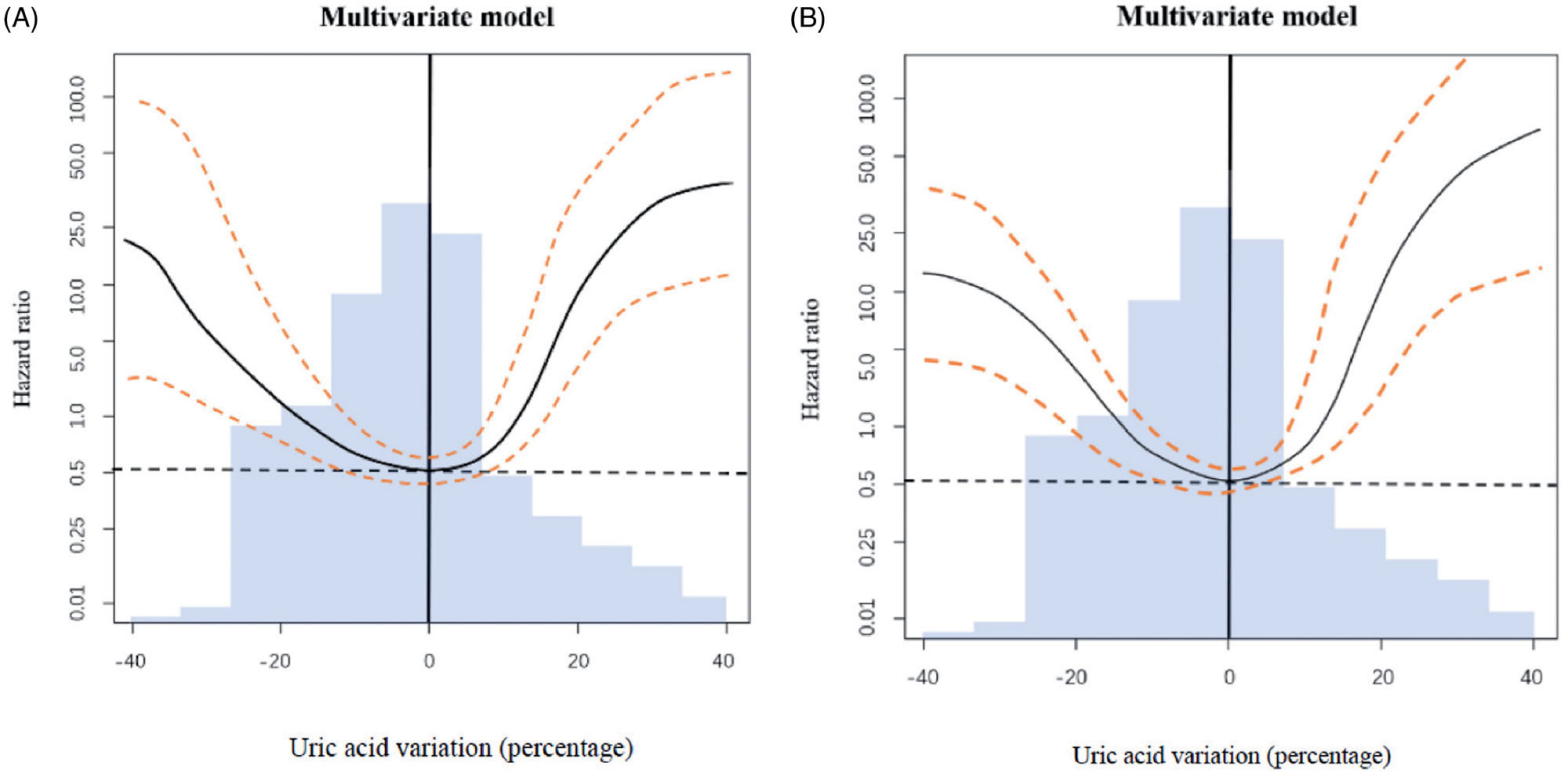
Serum UA changed from baseline and its association with relative risk of RRF loss. The change of serum UA from baseline was regarded as a time-varying variable. A change of 0 (no change) compared to baseline was used as the reference (hazard ratio = 1.0) in both graphs. The areas between two dashed lines showed serum values with minimum risk of residual renal function (RRF) loss. Multivariate model (A) included the following variables: peritonitis, total KT/V urea. Multivariate model(B) included the following variables: age, gender, urate-lowering drug use, peritonitis, and total KT/V urea.

Categorization of continuous variables may lead to loss of information, inaccurate estimation and difficulty in comparing results among different studies. To strengthen our findings, we further performed an analysis using Cox regression combined with penalized spline smoothing of time-varying UA levels (Figure 2). We observed a U-shaped relationship between time-varying UA levels and RRF loss. The greater in the change (increase or decrease) of UA level from baseline, the faster in decline of RRF (Figure 2).

## Discussion

The present study explored the relationship between UA level and RRF loss in CAPD patients. Our results indicated that time-averaged and time-varying serum UA levels are risk factors for RRF loss in patients receiving maintenance CAPD. We also found that both higher (≥7.64 mg/dL) and lower (<6.77 mg/dL) TA-UA levels were associated with an increased risk of RRF loss. It is suggested that serum UA during PD follow-up should be controlled within a relatively narrow range to slow the loss of RRF.

A number of studies have demonstrated that baseline serum UA level is important in ESRD patients, and that it is related to all-cause and cardiovascular mortality in dialysis patients. However, the baseline serum UA level only represents the situation before dialysis treatment; TA-UA is considered a more predictable and manageable parameter in ESRD patients. In CKD and dialysis patients, the effects of serum TA-UA level on cardiovascular outcomes, mortality, and progression of kidney function remain controversial or unknown. A higher level of TA-UA is considered to be the risk factor of CKD progression [16–18]. In addition, only a few studies have explored the effect of serum TA-UA on mortality in dialysis patients, but the results are inconsistent [19,20]. One recent study found that both higher and lower serum TA-UA levels increased all-cause mortality in PD patients [21]. RRF has been identified as a crucial predictor of outcomes in patients on maintenance dialysis. The beneficial effect of retaining RRF include better volume control, reduced number of complications, and improved quality of life [22–24]. However, only few studies have suggested that serum UA is a predictor of RRF loss in PD patients. The work of Park et al. [14] showed that hyperuricemia is common among PD patients, and that basal UA was significantly associated with a reduction of RRF. Another recent study found a U-shaped relationship between basal serum UA levels and RRF loss rate in patients on CAPD, with faster loss seen in both higher and lower UA groups [15]. However, these studies only measured baseline UA at a single time point and did not consider the overall trend of serum UA during follow-up. And TA-UA can reflect the change of UA level over time during PD follow-up. To avoid overlooking the effect of UA on clinical outcomes in survival analysis, we were the first to evaluate the association between TA-UA and time-varying UA and RRF loss. It was found that serum TA-UA and time-varying UA are independent risk factors for RRF loss in CAPD patients. Time-varying UA has a U-shaped, rather than linear, relationship with the risk of RRF loss.

Uric acid is considered a risk factor of CKD and ESRD in different populations, and UA has been reported to be associated with the loss of nephron even in normal individuals [8]. A high serum UA level is associated with oxidative stress, inflammatory response, endothelial dysfunction, development of metabolic syndrome, and activation of the renin-angiotensin system in CKD patients. Both *in vitro* and *in vivo* studies have shown that UA is an effective scavenger of oxygen-free radicals and a critical antioxidant. Total antioxidant capacity has been shown to correlate with serum UA levels in PD patients [25]. The antioxidative potential of serum UA may be beneficial in patients with increased oxidative stress. Therefore, reduction of serum UA level may reflect an inadequate compensatory response against oxidative damage, and suggests that variation in UA levels may facilitate a balance between its protective and harmful effects on kidney and cardiovascular outcomes. Conversely, increased loss of RRF in patients with TA-UA < 6.77 mg/dL might be related to malnutrition or insufficient protein intake, as UA levels have been associated with nutritional status. However, there were no significant differences in serum albumin, creatinine, phosphorous, hemoglobin, cholesterol, or body mass index among the three tertile groups in this study. Thus, other mechanisms may be involved in the impact of serum UA on prognosis.

Our study had several limitations. Firstly, it was a single-center, retrospective study. Thus causality could not be determined and potential presence of confounding factors could not be excluded. Secondly, the number of participants in our study was small, but meanwhile, the number of patients dropped out was relatively large. Thirdly, the study population was limited in patients who were treated with PD therapy, and the results may therefore not be applicable to patients on hemodialysis, or those with CKD. Despite these limitations, our studies indicated that TA-UA level is an independent predictor of RRF loss in PD patients, even after adjustment for demographic, clinical, laboratory, and dialysis-related variables.

In conclusion, we found that TA-UA is associated with RRF loss of CAPD patients and in a U-shaped relationship. An obvious increase or decrease in UA levels is associated with rapid declines in RRF. Uric acid level during follow-up is as important as baseline UA. Large-scale randomized controlled trials with longer follow-up are needed to identify the optimal UA level over time and determine the effect of urate lowering treatment on progression of RRF in PD patients.

## References

[1] Harrison R. Structure and function of xanthine oxidoreductase: Where are we now? Free Radic. Biol. Med. 2002;33(6):774–797. 12208366 10.1016/s0891-5849(02)00956-5

[2] Sautin YY, Johnson RJ. Uric acid: the oxidant-antioxidant paradox. Nucleos Nucleot Nucleic Acids. 2008;27(6-7):608–619. 10.1080/15257770802138558 PMC2895915 18600514

[3] Domrongkitchaiporn S, Sritara P, Kitiyakara C, et al. Risk factors for development of decreased kidney function in a southeast Asian population: a 12-year cohort study. JASN. 2005;16(3):791–799. 15677313 10.1681/ASN.2004030208

[4] Obermayr RP, Temml C, Gutjahr G, et al. Elevated uric acid increases the risk for kidney disease. JASN. 2008;19(12):2407–2413. 18799720 10.1681/ASN.2008010080 PMC2588108

[5] Weiner DE, Tighiouart H, Elsayed EF, et al. Uric acid and incident kidney disease in the community. JASN. 2008;19(6):1204–1211. 18337481 10.1681/ASN.2007101075 PMC2396939

[6] Hsu CY, Iribarren C, McCulloch CE, et al. Risk factors for end-stage renal disease: 25-year follow-up. Arch Intern Med. 2009;169(4):342–350. 19237717 10.1001/archinternmed.2008.605 PMC2727643

[7] Chonchol M, Shlipak MG, Katz R, et al. Relationship of uric acid with progression of kidney disease. Am. J. Kidney Dis. 2007;50(2):239–247. 17660025 10.1053/j.ajkd.2007.05.013

[8] Denic A, Mathew J, Lerman LO, et al. Single-nephron glomerular filtration rate in healthy adults. N Engl J Med. 2017;376(24):2349–2357. 28614683 10.1056/NEJMoa1614329 PMC5664219

[9] Madero M, Sarnak MJ, Wang X, Greene T, et al. Uric acid and long-term outcomes in CKD. Am J Kidney Dis. 2009;53(5):796–803. 19303683 10.1053/j.ajkd.2008.12.021 PMC2691553

[10] Ohno I, Hosoya T, Gomi H, et al. Serum uric acid and renal prognosis in patients with IgA nephropathy. Nephron. 2001;87(4):333–339. 11287777 10.1159/000045939

[11] Srivastava A, Kaze AD, McMullan CJ, et al. Uric acid and the risks of kidney failure and death in individuals with CKD. Am J Kidney Dis. 2018;71(3):362–370. 29132945 10.1053/j.ajkd.2017.08.017 PMC5828916

[12] De Cosmo S, Viazzi F, Pacilli A, et al.; the AMD-Annals Study Group. Serum uric acid and risk of CKD in type 2 diabetes. CJASN. 2015;10(11):1921–1929. 26342044 10.2215/CJN.03140315 PMC4633786

[13] Tsai CW, Chiu HT, Huang HC, et al. Uric acid predicts adverse outcomes in chronic kidney disease: a novel insight from trajectory analyses. Nephrol Dial Transplant. 2018;33(2):231–241. 29140472 10.1093/ndt/gfx297

[14] Park JT, Kim DK, Chang TI, et al. Uric acid is associated with the rate of residual renal function decline in peritoneal dialysis patients. Nephrol Dial Transplant. 2009;24(11):3520–3525. 19491381 10.1093/ndt/gfp272

[15] Hsieh YP, Yang Y, Chang CC, et al. U-shaped relationship between uric acid and residual renal function decline in continuous ambulatory peritoneal dialysis patients. Nephrology (Carlton). 2017;22(6):427–435. 26370323 10.1111/nep.12613

[16] Chang WX, Xu N, Kumagai T, et al. Uric acid in the follow up determines 30% decline in estimated gfr over 2 years: a propensity score analysis. Kidney Blood Press Res. 2017;42(6):1053–1067. 29346798 10.1159/000485593

[17] Uchida S, Chang WX, Ota T, et al. Targeting uric acid and the inhibition of progression to end-stage renal disease—a propensity score analysis. PLoS One. 2015;10(12):e0145506. 26700005 10.1371/journal.pone.0145506 PMC4689349

[18] Shu D, Xu F, Su Z, et al. Risk factors of progressive IgA nephropathy which progress to end stage renal disease within ten years: a case–control study. BMC Nephrol. 2017;18(1):11. 28061828 10.1186/s12882-016-0429-x PMC5219698

[19] Bae E, Cho HJ, Shin N, et al. Lower serum uric acid level predicts mortality in dialysis patients. Medicine (Baltimore). 2016;95(24):e3701. 27310949 10.1097/MD.0000000000003701 PMC4998435

[20] Dong J, Han QF, Zhu TY, Ren YP, et al. The associations of uric acid, cardiovascular and all-cause mortality in peritoneal dialysis patients. PLoS One. 2014;9(1):e82342. 24416142 10.1371/journal.pone.0082342 PMC3885378

[21] Miller BW, Himmele R, Sawin DA, et al. The association of longitudinal serum uric acid and all-cause mortality in incident peritoneal dialysis patients. Blood Purif. 2018;45(1-3):224–229. 30463062 10.1159/000494987

[22] Perl J, Bargman JM. The importance of residual kidney function for patients on dialysis: a critical review. Am J Kidney Dis. 2009;53(6):1068–1081. 19394737 10.1053/j.ajkd.2009.02.012

[23] Termorshuizen F, Korevaar JC, Dekker FW, et al. The relative importance of residual renal function compared with peritoneal clearance for patient survival and quality of life: an analysis of the Netherlands Cooperative Study on the Adequacy of Dialysis (NECOSAD)-2. Am J Kidney Dis. 2003;41(6):1293–1302. 12776283 10.1016/s0272-6386(03)00362-7

[24] Termorshuizen F, Dekker FW, van Manen JG, et al. Krediet RT; NECOSAD Study Group. Relative contribution of residual renal function and different measures of adequacy to survival in hemodialysis patients: an analysis of the Netherlands Cooperative Study on the Adequacy of Dialysis(NECOSAD)-2. J Am Soc Nephrol. 2004;15(4):1061–1070. 15034110 10.1097/01.asn.0000117976.29592.93

[25] Kim SB, Yang WS, Min WK, et al. Reduced oxidative stress in hypoalbuminemic CAPD patients. Perit Dial Int. 2000;20(3):290–294. 10898045

